# PD-1 limits differentiation and plasticity of Tc17 cells

**DOI:** 10.3389/fimmu.2023.1104730

**Published:** 2023-04-28

**Authors:** Aditya Arra, Holger Lingel, Mandy Pierau, Monika C. Brunner-Weinzierl

**Affiliations:** ^1^Department of Experimental Pediatrics, University Hospital, Otto-von-Guericke-University, Magdeburg, Germany; ^2^Health Campus Immunology, Infectiology and Inflammation, Otto-von-Guericke-University, Magdeburg, Germany

**Keywords:** T-cell differentiation, T-cell plasticity, Tc17 cells, cytotoxic T lymphocytes (CTLs), immune checkpoint

## Abstract

Blockade of surface co-inhibitory receptor programmed cell death-1 (PD-1; CD279) has been established as an important immunotherapeutic approach to treat malignancies. On a cellular level, PD-1 is demonstrated to be of particular importance in inhibiting differentiation and effector function of cytotoxic Tc1 cells (CTLs). Nevertheless, the role of PD-1 in modulating interleukin (IL)-17-producing CD8^+^ T-cells (Tc17 cells), which generally display suppressed cytotoxic nature, is not well understood. To evaluate the impact of PD-1 in Tc17 responses, we examined its functioning using different *in vitro* and *in vivo* models. Upon activation of CD8^+^ T-cells in Tc17 environment, we found that PD-1 was rapidly expressed on the surface of CD8^+^ T-cells and triggered a T-cell-internal mechanism that inhibited the expression of IL-17 and Tc17-supporting transcription factors pSTAT3 and RORγt. Expression of type17-polarising cytokine IL-21 and the receptor for IL-23 were also suppressed. Intriguingly, adoptively transferred, PD-1^-/-^ Tc17 cells were highly efficient in rejection of established B16 melanoma *in vivo* and displayed Tc1 like characteristics *ex vivo*. When using IL-17A-eGFP reporter mice for *in vitro* fate tracking, IL-17A-eGFP expressing cells lacking PD-1 signaling upon re-stimulation with IL-12 quickly acquired Tc1 characteristics such as IFN-γ, and granzyme B expression, implicating lineage independent upregulation of CTL-characteristics that are needed for tumor control. In line with plasticity characteristics, absence of PD-1 signaling in Tc17 cells increased the expression of the stemness and persistence-associated molecules TCF1 and BCL6. Thus, PD-1 plays a central role in the specific suppression of Tc17 differentiation and its plasticity in relation to CTL-driven tumor rejection, which provides further explanation as to why the blockade of PD-1 is such an efficient therapeutic target for inducing tumor rejection.

## Introduction

Effector CD8^+^ T-cells that eliminate infected and malignant cells in an antigen (Ag)-specific manner play a crucial role in host protection. Upon encounter with endogenous antigens on antigen-presenting cells (APCs), either from intracellular pathogens or tumors, naïve CD8^+^ T-cells become activated and differentiate into effector cytotoxic T lymphocytes (1, 2). CTLs expressing cytokines IL-2, interferon (IFN)-γ and tumor necrosis factor-alpha (TNF-α), also referred as Tc1 cells, are well defined and are endowed with high cytotoxic capacity and destroy their targets by releasing cytotoxic molecules such as perforin and granzymes into the immunological synapse (3, 4). Depending on the activation conditions and cytokine microenvironment, CD8^+^ T-cells can also differentiate into IL-17-producing subset called Tc17 cells. A microenvironment containing transforming growth factor β (TGF-β) and IL-6 drive differentiation of Tc17 cells. These cells express high levels of IL‐17, low IFN‐γ, and demonstrate a limited cytotoxic activity lacking granzyme B expression (5). Tc17 cells participate in inflammatory processes and play a role in several infectious and autoimmune diseases (6–8) as well as several types of human tumors including gastric, uterus, head and neck, liver, and colon cancer (9–13).

Despite the fact that *in vitro*-generated Tc17 cells have a limited cytotoxic activity, it has been reported that adoptive transfer of these cells promotes the elimination of established tumors in mice (14, 15). Additionally, Tc17 cells have been shown to provide host immunity against viral infections such as influenza and vaccinia virus (16, 17). These contradictory abilities of Tc17 cells are due to their microenvironment‐dependent lineage plastic nature. Highly plastic Tc17 cells convert toward a Tc1‐like phenotype upon transfer into tumor-bearing mice while maintaining some of their Tc17 characteristics, such as cell longevity (18, 19). Moreover, it has been suggested that Tc17 cells exhibits protective functionality *in vivo* in an IFN-γ-dependent manner (14, 20). However, factors that determine Tc17 lineage plasticity or stability, though not fully understood so far, remain of major interest to improve antitumor therapies.

The inhibitory receptor PD-1, expressed mostly by activated T-cells acts as a negative regulator of the immune response. PD-1 plays a major role in CD8^+^ T-cell exhaustion during chronic infections and cancer (21–23). Engagement of PD-1 by its ligands (PD-L1 and PD-L2) conveys negative signals through immunoreceptor tyrosine-based switch motif (ITSM) recruitment of SH2-domain–containing tyrosine phosphatase (SHP-2) that dephosphorylates initial stimulatory signaling molecules (24). It was also recently reported that PD-1 not only inhibits T cell receptor (TCR) signaling but also targets the costimulatory molecule CD28 (25, 26). The discovery of the fundamental role of PD-1 led to the idea that PD-1 signaling could be altered to help the immune system fight cancer or infections. It has been reported in several animal models that blockade of PD-1 inhibitory pathways breaks the immune tolerance and leads to activation of CTLs and results in reduction of viral load and better control of tumor progression (23, 27–29). In addition, PD-1–directed immunotherapy has shown clinical efficacy in human cancers, and PD-1 pathway inhibitors are now licensed for the treatment of a wide variety of cancers (30–36). Focusing on the major effector cells that eliminate tumors and infections, PD-1–directed immunotherapy has been shown to enhance cytotoxic activity of CD8^+^ T-cells by increasing IFNγ and granzyme B production.

PD-1 is also expressed during the early phase of T-cell activation when naive CD8^+^ T-cells differentiate into effector cells. It has been reported that this transient PD-1 expression plays a regulatory role during CD8^+^ T-cell differentiation and it’s blockade is demonstrated to be of particular importance in enhancing differentiation and effector function of IFNγ and granzyme B-producing Tc1 cells (37). Even though the impact of PD-1 on Tc1 differentiation and effector responses is well appreciated, its role in Tc17 biology is not well defined.

In this study we address the role of PD-1 in Tc17 cells and report that PD-1 plays a negative regulatory role during naïve to effector differentiation of IL-17-producing CD8^+^ T-cells. Activation of OT-1 PD-1^+/+^ CD8^+^ T-cells or CD8^+^ T-cells in the presence of PD-1 engagement simultaneously with anti-CD3 and anti-CD28 showed a reduced cellular ability to execute the Tc17 program. Conversely, PD-1^-/-^ OT-1 CD8^+^ T-cells showed an augmented capacity to enhance Tc17 differentiation and mount immunity to tumor progression while producing IFN-γ upon adoptive transfer into tumor bearing mice. PD-1 signaling not only reduced IL-17, IL-23 receptor (IL-23R), IL-21 and retinoic acid receptor-related orphan receptor gamma t (RORγt) expression in Tc17 cells but also dampened stemness associated T cell factor 1 (TCF1) and plastic nature of Tc17 cells to convert to Tc1 like cells and thereby their cytotoxic activity. Together, these findings add a novel mechanism to the knowledge of how PD-1 regulates CD8^+^ T-cell immunity and thus, opens new ways for therapeutic intervention in malignancies.

## Materials and methods

### Mice and cell line

PD-1^+/+^ OT-1 and PD-1^-/-^ OT-1 mice, expressing a transgenic TCR (TCR^tg^) specific for ovalbumin (OVA) peptide residue 257-264:H2K^b^ and as well as PD-1^-/-^, Ly5.1, C57BL/6 and IL-17A- enhanced green fluorescent protein (eGFP) reporter mice were bred under specifically pathogen free conditions following institutional guidance, at the central animal facility of University of Magdeburg Medical School (Germany). PD-1^-/-^ mice on C57BL/6 background were kindly provided by Prof. Luisa Klotz, Muenster, Germany, and were crossbred with OT-1 mice to generate PD-1^-/-^ OT-1 mice. IL-17A-eGFP reporter mice were kindly provided by Prof. Anja E. Hauser, Berlin, Germany. All animal experiments were performed in accordance to the institutional and state guidelines. The OVA-transfected B16 tumor cell line (B16-OVA) was kindly provided by Prof. Karl Sebastian Lang, Essen, Germany. Cells were maintained in RPMI 1640, supplemented with 10% heat-inactivated fetal-calf serum (FCS), 25mM HEPES, 1mM Sodium pyruvate (Sigma Aldrich), 50µM 2-mercaptoethanol and 1 mg/ml G418 (Carl Roth).

### T-cell differentiation

Naïve CD8^+^ T-cells (CD8^+^ CD62L^high^) from spleen, inguinal, axillary and mesenteric lymph nodes of PD-1^+/+^ and PD-1^-/-^ OT-1 mice were isolated by magnetic beads separation using AutoMACSpro (Miltenyi Biotec). Comparable levels of CD25 and CD69 expression were routinely determined using flow cytometry (data not shown). For antigen (Ag)-specific activation, OT-1 CD8^+^ T-cells were stimulated with 0.25 μg/ml of endotoxin-free SIINFEKL (OVA257-264) peptide (Invivogen) and CD90-depleted splenocytes from C57BL/6 mice at a 1:6 ratio. To determine strength of TCR signal in T-cell differentiation, OVA peptide variants of different concentrations and different affinities (SIINFEKL, Invivogen; SIITFEKL, Eurogentec) were used. For antibody specific agonistic stimulation, CD8^+^ T-cells isolated from spleen, inguinal, axillary and mesenteric lymph nodes of C57BL/6 mice were used. The isolated cells were stimulated with antibodies immobilized on microspheres. The total amount of protein was kept at 5µg per 10^7^ microspheres which were immobilized with 1µg anti-CD3 (145-2C11) (20% of total), 1 µg of anti-CD28 (37.51) (Miltenyi Biotec) and 60% of either recombinant PD-L1 (rPD-L1) (R&D systems) or IgG1. All cells were cultured in serum free x-vivo 15 medium (Lonza). For Tc17 differentiation the cells were conditioned with 2 ng/ml TGF-β, 10 ng/ml IL-6 (R&D systems), 10 ng/ml IL-23 (Biolegend) with or without 5 μg/ml anti-IFN-γ XMG.1.2 (Biolegend) or anti-IL-2 (S4B6) (BD Biosciences). To determine Tc17 plasticity, primary anti-CD3, anti-CD28 (coupled to microspheres) stimulated Tc17 cells were re-stimulated with anti-CD3, anti-CD28, and/or rPD-L1 antibodies immobilized to microspheres and were conditioned with 5 ng/ml IL-12 and 5 ng/ml IL-2.

### Antibodies and flow cytometry

Antibodie (Ab)s used in flow cytometric analysis are listed in **Supplementary Table S1**. Expression of cell surface molecules CD8α, CD25, CD44, CD45.2, CTLA-4, CD69, IL-6Rα, IL-23R, ICOS, PD-1, PD-L1 etc. was detected on the cells by flow cytometry. For this purpose, the cells were harvested and pelleted by brief centrifugation and stained with specific antibodies listed in **Table S1** in phosphate-buffered saline (PBS)/0.2% bovine serum albumin (BSA) for 10 min at 4°C and measured by flow cytometry. Cell surface CD107a was measured using flow cytometry by staining in culture medium with phorbol myristate acetate (PMA), ionomycin and Brefeldin A for 4 h at 37°C. For detection of intracellular cytokines IL-17, IFN-γ, IL-2, IL-21, TNF-α etc. the cells were re-stimulated with PMA, ionomycin and Brefeldin A for 4 h at 37°C. The cells were then harvested and pelleted by brief centrifugation, and stained for surface molecules for 10 min at 4°C followed by fixation with 2% paraformaldehyde (Merck) in PBS for 20 min and permeabilized in 0.5% saponin (Sigma-Aldrich) in PBS/BSA. The cells were then stained for cytokines with the specific antibodies listed in **Table S1** in 0.5% saponin (in PBS/BSA) for 20 min at 4°C and measured using flow cytometry in PBS/BSA. In some experiments, cells were labelled with vital dyes carboxyfluorescein succinimidyl ester (CFSE) or cellTrace violet (CTV) (Thermo Fisher Scientific) protected from light and washed twice prior to stimulation. The expression of eomesodermin (Eomes), B-cell lymphoma 6 (BCL6), TCF1, phosphorylated signal transducer and activator of transcription 3 (pSTAT3) (10min pretreatment with IL-6+IL-23 before cell harvesting), RORγt, Granzyme B etc. was also measured by flow cytometry. For this purpose, the cells were harvested and briefly pelleted by centrifugation, and fixed in 4% formaldehyde (Carl Roth) in PBS for 10 min at 37°C, followed by permeabilization in ice-cold 90% methanol for 30 min (Carl Roth). The cells were then stained in PBS/BSA for 60 min at room temperature using antibodies listed in **Table S1** and measured using flow cytometry. Variations in flow cytometry (FACS) analyses were normalized. All cytometric measurements were performed using a FACS-Canto II (BD Biosciences) and analyzed with FlowJo software (FlowJo LLC). The gating strategy for flow cytometric analyses is demonstrated in the **Supplementary Figure S1**.

### Adoptive T-cell transfer and melanoma model

CD45.1 (Ly5.1) mice were subcutaneously (s.c.) injected with 2x10^5^ B16-OVA melanoma cells. Mice that had developed a palpable tumor received an i.v. injection with either PBS or 1 x 10^6^ *in vitro* generated CD45.2-expressing PD-1^+/+^ or PD-1^-/-^ OT-1 Tc17 cells (CD8^+^ T-cells stimulated for 3 days under Tc17 condition) on day 8 after the tumor cell injection. Tumor growth was then monitored and upon development of large tumors, the mice were humanely sacrificed, and the experiment was terminated. Following that, adoptively transferred CD8^+^ CD45.2^+^ cells were identified and analyzed ex vivo in single cell suspensions of spleen and tumor draining lymph nodes of tumor bearing mice by flow cytometry.

### Real-time polymerase chain reaction

Using Tc17 stimulated total CD8^+^ T-cells (from C57BL/6 mice) at indicated times, RNA was isolated from pelleted cells with NucleoSpin RNA Isolation Kit (Macherey-Nagel). The cDNA was synthesized using revertAid H minus first strand cDNA synthesis kit (Thermo Fisher Scientific) and stored at –20°C. Primer pairs to investigate the gene expression profile of RORc, BCL6, TCF1 (TCF7), T-box transcription factor (T-bet), hypoxia inducible factor 1 alpha (HIF-1α), interferon regulatory factor 4 (IRF4), IL-23R, IL-21 and IL-17a by real-time PCR (quantative (q) PCR) were purchased from TIB MOLBIOL (primer sequences are shown in **Supplemental Table S2**). Gene expression was analyzed using Maxima SYBR Green qPCR Master Mix (Thermo Scientific) on a CFX96 Real-Time PCR detection system (Bio-Rad). Fold change in the expression of target genes was normalized to the expression of housekeeping gene GAPDH.

### *In vitro* CD8^+^ T-cell cytotoxicity assay

In order to measure antigen-specific CD8^+^ T-cell killing efficiency an *in vitro* cytotoxicity assay was performed. For this assay, T-cell-depleted splenocytes were used, which were labeled separately, with two different concentrations of vital dye CFSE (5 μM CFSE (CFSE^high^ cells) or 0.25 μM CFSE (CFSE^low^ cells)) for 5 min and washed twice with x-vivo 15 medium. CFSE^high^ cells were pulsed with the antigen OVA_257–264_-peptide (1 hr, 37°C) and were considered as target cells for OVA_257–264_-specific CD8^+^ T-cells. CFSE^low^ cells, which were not pulsed with OVA-peptide, were used as controls. OVA_257–264_-pulsed (CFSE^high^) and -unpulsed (CFSE^low^) T-cell depleted splenocytes were mixed at a 1:1 ratio and were cultured together with three days predifferentiated OVA_257–264_-specific PD-1^+/+^ or PD-1^−/−^ OT-1 Tc17 cells at 1:6 ratio. After 24 hours, CFSE-labeled cells were detected and quantified using flow cytometry and percent of lysis of target cells was calculated by difference between CFSE^low^ versus CFSE^high^ cells.

### Statistics

Data were analyzed by Microsoft Excel 2010 (Microsoft Co., USA) and Graph Pad Prism 8 (Graphpad software Inc, USA). Data are presented as mean ± SD. P-values were computed by using unpaired or Welch’s t-test. Multiple comparisons between more than two independent groups were performed using two-way ANOVA with repeated measures (RM) followed by Holm-Sidak’s multiple comparisons test for each individual time point. Statistical significance is indicated as follows: ****P < 0.0001, ***P < 0.001, **P < 0.01, *P < 0.05, n.s: not significant.

## Results

### PD-1 restricts differentiation of IL-17-producing CD8^+^ T-cells

**Figure 1:**
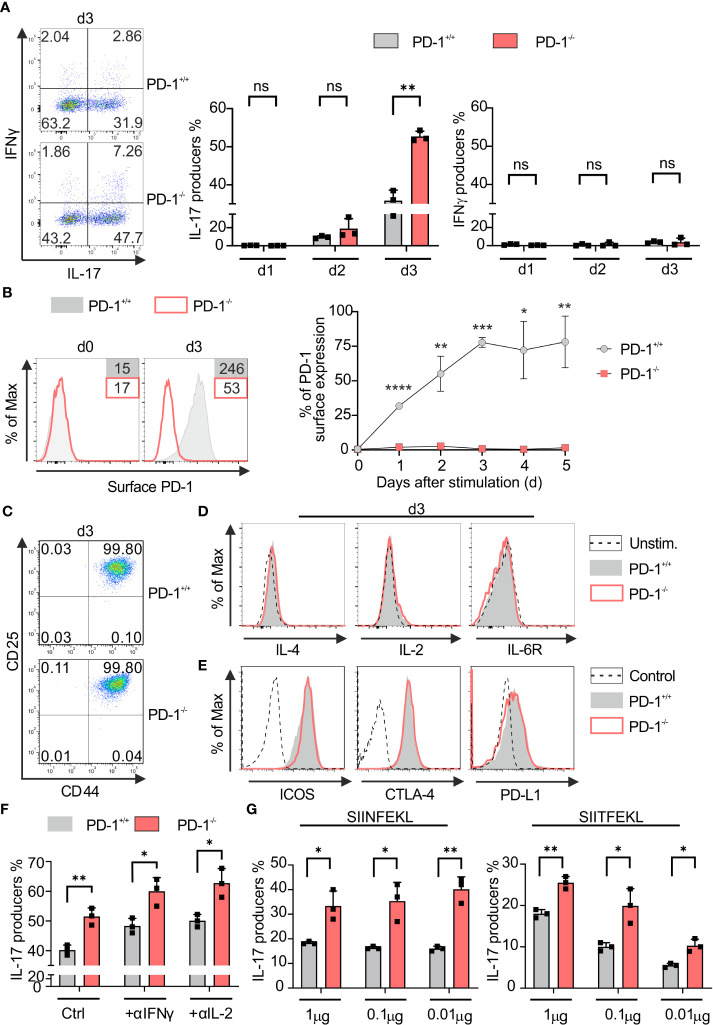
PD-1 restricts Tc17 differentiation. PD-1^+/+^ and PD-1^-/-^ OT-1 naïve CD8^+^ T cells were activated antigen specifically using OVA_257–264_ loadedAPCs and cultured under Tc17 conditions. **(A)** Dot plot representing flow cytometric analysis of intracellular IL-17 and IFN-γ expression (left panel) in OT-1 CD8^+^ T cells stimulated for 3 days under Tc17 conditions. Cumulative staining results from d1, d2 and d3 are shown on the right. **(B)** Histogram of PD-1 surface expression 3 days after antigen specific stimulation of PD-1^+/+^ Tc17 cells. PD-1^-/-^ Tc17 cells were used as negative controls. Graph representing kinetics of PD-1 expression on surface of PD-1^+/+^ and PD-1^-/-^ Tc17 cells at different time points from day 0 to day 5. **(C, D)** Naïve PD-1^+/+^ and PD-1^-/-^ OT-1 CD8^+^ T cells were stimulated as in **(A)** for 3 days and analyzed for the expression of activation markers CD25 and CD44 **(C)**, intracellular cytokines IL-4, IL-2, cytokine receptors IL-6R **(D)** by flow cytometry. **(E)** Histograms of surface expression of ICOS, CTLA-4 and PD-L1 in 3 day stimulated PD-1^+/+^ and PD-1^-/-^ Tc17 cells. **(F)** Naïve PD-1^+/+^ and PD-1^-/-^ OT-1 CD8^+^ T cells were stimulated as in **(A)** and intracellular IL-17 expression with and without neutralizing antibodies against IL-2 and IFN-γ was measured by flow cytometry on day3 after activation and presented in the bar graph. **(G)** Naïve PD-1^+/+^ and PD-1^-/-^ OT-1 CD8^+^ T cells were stimulated with different concentrations (1μg, 0. 1μg and 0.01μg) of high affinity (SIINFEKL) and low affinity (SIITFEKL) OVA peptide loaded APCs under Tc17 conditions. Bar graphs represent cumulative frequencies of intracellular IL-17 producers 3 days after activation. The data are representative of two to three independent experiments. Data points represent individual experiments with mean+SD. **** P < 0.0001, ***P < 0.001, **P < 0.01, *P < 0.05, n.s: not significant, calculated by Welch’s *t-*test.

PD-1 signaling subdues IFN‐γ‐production and granzyme B secretion of Tc1 cells (37, 38). In order to determine the PD-1-mediated impact during the differentiation of Tc17 cells, we first applied a model for Tc17 differentiation, therefore, we stimulated naive PD-1^−/−^ and PD-1^+/+^ OT-1 CD8^+^ T-cells with OVA_257–264_ peptide and congenic APCs in Tc17‐skewing conditions (IL-6, IL-23, TGFβ, see *Materials and methods*). Enriched PD-1^−/−^ and PD-1^+/+^ OT-1 CD8^+^ T-cells were routinely controlled having a naïve phenotype using expression of activation‐regulated molecules and effector cytokine expression (data not shown). Strikingly, even under Tc17‐skewing conditions, PD-1 had a suppressive effect on effector cytokine production. PD-1^−/−^ Tc17 cells showed a significantly high frequency of IL‐17 producers than PD-1^+/+^ Tc17 cells three days after beginning of the stimulation (**Figure 1A**, middle panel). In accordance with other studies (5), under Tc17 conditions without IFN‐γ neutralization, IFN‐γ production was visible at low frequencies (**Figure 1A**, left panel). Even though IFN‐γ is known to negatively affect differentiation of IL-17-producing cells (39, 40), a marginally enhanced frequency of IFN‐γ producers within PD-1^−/−^ Tc17 cells had no suppressive effect on enhanced Tc17 differentiation (**Figure 1A**). To determine the kinetics upon activation of CD8^+^ T-cells in a Tc17 milieu to up-regulate PD-1 at the cell surface, primary stimulation of OT-1 CD8^+^ T-cells under Tc17‐skewing conditions was performed. Within 24hrs of the onset of stimulation, PD-1^+/+^ Tc17 cells upregulated PD-1 on their surface with highest frequency of expression occurring on day three (**Figure 1B**) which explains the strong effect of PD-1 on Tc17 differentiation. Since all T-cells of both PD-1^−/−^ and PD-1^+/+^ Tc17 cell types express the activation-induced surface molecules CD44 and CD25, differences in activation are unlikely to be the cause of differential IL-17 production (**Figure 1C**). In the same line, expression of the cytokines IL-10, TNF-α (data not shown), IL-2 and IL-4 remained similar in both PD-1^+/+^ and PD-1^−/−^ Tc17 cell types (**Figure 1D**). In addition, even surface expression of co-receptors cytotoxic T lymphocyte antigen 4 (CTLA-4), inducible T-cell co-stimulator (ICOS) and PD-L1, which are otherwise known to regulate differentiation of IL-17-producing cells (41–44), was observed to be similar in both PD-1^+/+^ and PD-1^−/−^ Tc17 cell types (**Figure 1E**), as was IL-6R. These results indicated that compared with PD-1 competent CD8^+^ T-cells, despite showing similar upregulation of activation-induced molecules and of co- and inhibitory molecules, PD-1^−/−^ CD8^+^ T-cells specifically induce higher frequencies of IL‐17^+^ cells under Tc17 conditions.

To exclude the strong inhibitory effects of Type-1 cytokines on PD-1 regulated Tc17 differentiation (39), we compared IL-17 expression by PD-1^+/+^ and PD-1^−/−^ Tc17 population with or without neutralizing antibody against IFN‐γ or IL-2, respectively. However, despite neutralization, PD-1^−/−^ Tc17 cells still displayed enhanced frequencies of IL-17 producers in comparison to that of PD-1^+/+^ (**Figure 1F**). As strength of TCR signals affects T‐cell differentiation (45) and tumor antigens might often be of low affinity due to their autoantigenic nature (46), low affinity stimulation was initiated using SIITFEKL peptides for OT-1 TCRtg T-cells besides high affinity stimuli (SIINFEKL) with a wide range of concentrations as indicated (**Figure 1G**). Regardless of the affinity used, genetic inactivation of PD-1 in CD8^+^ T-cells was superior to PD-1-competent CD8^+^ T-cells in mediating increased abundance of IL-17 producers (**Figure 1G**), which may be a double-edged sword for antitumor responses. On the one hand, Tc17 responses that may not be cytotoxic are enhanced; on the other hand, under many conditions, tumor antigen-specific T-cell responses may even be enhanced against low-affinity tumor antigens under PD-1 blockade.

### PD-1-mediated suppression of Tc17 differentiation is T-cell intrinsic

**Figure 2:**
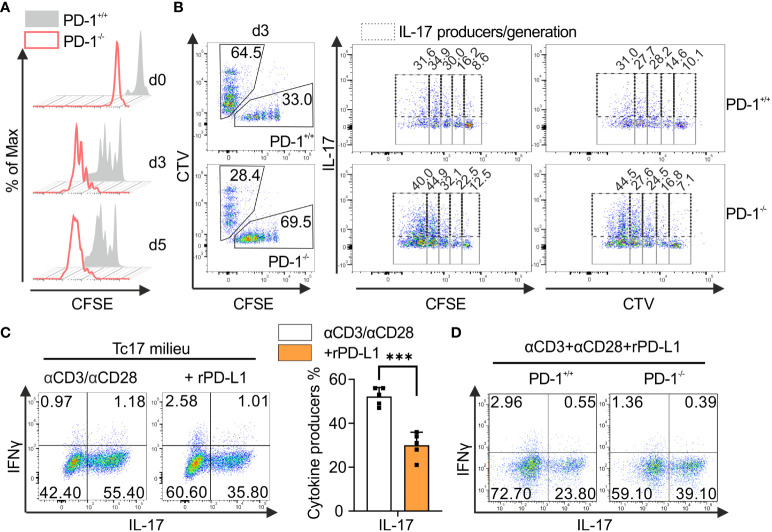
PD-1-mediated cell intrinsic signaling regulates Tc17 differentiation. **(A)** Naïve PD-1^+/+^ and PD-1^-/-^ OT-1 CD8^+^ T cells were labelled with CFSE (5μM) before priming them with OVA_257–264_ loaded APCs and cultured under Tc17 conditions. Proliferation of PD-1^+/+^ and PD-1^-/-^ Tc17 cells was evaluated by CFSE dilution at indicated time points. **(B)** Naïve PD-1^+/+^ and PD-1^-/-^OT-1 CD8^+^ T cells were labelled with CFSE (5μM) or CTV (2.5μM) or vice versa. CFSE and CTV stained cells were combined 1:1 prior to adding OVA_257–264_ loaded APCs. At day 3 after primary activation under Tc17 conditions cells were analyzed by flow cytometry for expression of IL-17. Dot plot representing IL-17 expression (dashed gates) in PD-1^+/+^ and PD-1^-/-^ Tc17 cells within each generation (solid gates) of proliferating cells. **(C)** CD8^+^ T-cells from C57BL/6 mice were stimulated with microspheres immobilized with anti-CD3, anti-CD28 and rPD-L1 (+rPD-L1) or IgG (αCD3/αCD28) under Tc17 polarizing conditions. Three days after primary stimulation, IL-17 and IFN-γ expression in these cells was measured by flow cytometry and are presented in the bar graph. **(D)** CD8^+^ T-cells from PD-1^+/+^ and PD-1^-/-^ mice were stimulated with microspheres immobilized with anti-CD3, anti-CD28 and rPD-L1 under Tc17 conditions and at day three IL-17 and IFN-γ expression in these cells was measured by flow cytometry. The data is representative of two to five independent experiments. Data points represent individual experiments with mean+SD. ***P<0.001, calculated by Welch’s *t-*test.

To better understand the role of PD-1 inactivation in Tc17 responses and to exploit this for tumor therapy, PD-1-mediated effects were next analyzed to determine whether the regulation of Tc17 differentiation was either due to increased differentiation or simply to increased proliferation (47). To determine proliferation-control of CD8^+^ T-cells by PD-1 under Tc17 differentiation, PD-1^+/+^ and PD-1^−/−^ OT-1 CD8^+^ T-cells were labelled with vital fluorescent dye CFSE before primary stimulation under Tc17‐skewing conditions, thus, being able to monitor each single cell cycle generation by fluorescence loss. Our results indicate that in absence of PD-1 signaling Tc17 cells displayed overall enhanced proliferation determined by faster mitotic events and enhanced frequencies of original T-cells (G_0_ at beginning of the stimulation) entering cell cycle (**Figure 2A**). To determine whether soluble factors in the microenvironment or the APCs regulate PD-1-mediated suppression of Tc17 differentiation and proliferation, we performed a co-culture experiment where equal amounts of enriched PD-1^+/+^ and PD-1^−/−^ OT-1 CD8^+^ T-cells were mixed and stimulated together with OVA_257–264_ loaded APCs in a Tc17 cytokine milieu (**Figure 2B**). Since TGFβ a strong survival factor was used in Tc17 differentiation, cell death was negligible (48). To distinguish between the different cell populations, PD-1^+/+^ or PD-1^−/−^ OT-1 CD8^+^ T-cells were stained with vital fluorescent dyes CFSE or CTV or vice versa prior to activation. Our data unambiguously show that PD-1^−/−^ CD8^+^ T-cells generated higher frequencies of Tc17 cells exceeding that of PD-1^+/+^ CD8^+^ T-cells even within the same microenvironment. In addition, frequency of IL-17 expressing cells within fast proliferating cells was also enhanced in PD-1^−/−^ Tc17 cells in comparison to PD-1^+/+^ ones (**Figure 2B**). Thus, PD-1 controls proliferation of Tc17 cells and their differentiation, both effects by a cell intrinsic mechanism. To strengthen our finding, we used an experimental approach that provided similar amounts of CD3- and CD28-triggering, and excluded further extrinsic signals from B7 ligands on APCs. In this approach, wild type mouse CD8^+^ T-cells were stimulated with microspheres coupled with anti-CD3, anti-CD28 plus either rPD-L1 or IgG control. CD8^+^ T-cells that were engaged with rPD-L1 (anti-CD3, anti-CD28 +rPDL1) displayed a significant reduction in the frequency of IL-17-producing cells compared with Tc17 cells engaging CD3 and CD28 only (**Figure 2C**). Similarly, enhanced frequency of IL-17 producers was also observed in CD8^+^ T-cells from PD-1^-/-^ mice that were stimulated by crosslinking with anti-CD3, anti-CD28 and rPD-L1 in comparison to PD-1^+/+^ cells (**Figure 2D**). Together, these data demonstrate that the suppressive effect of PD-1 on Tc17 differentiation is unambiguously cell‐intrinsic and independent of differential APC activation or extrinsic, soluble factors in the microenvironment.

### Lack of PD-1 signaling enhances cytokine and transcription factor profile characteristics of Tc17 cells

**Figure 3:**
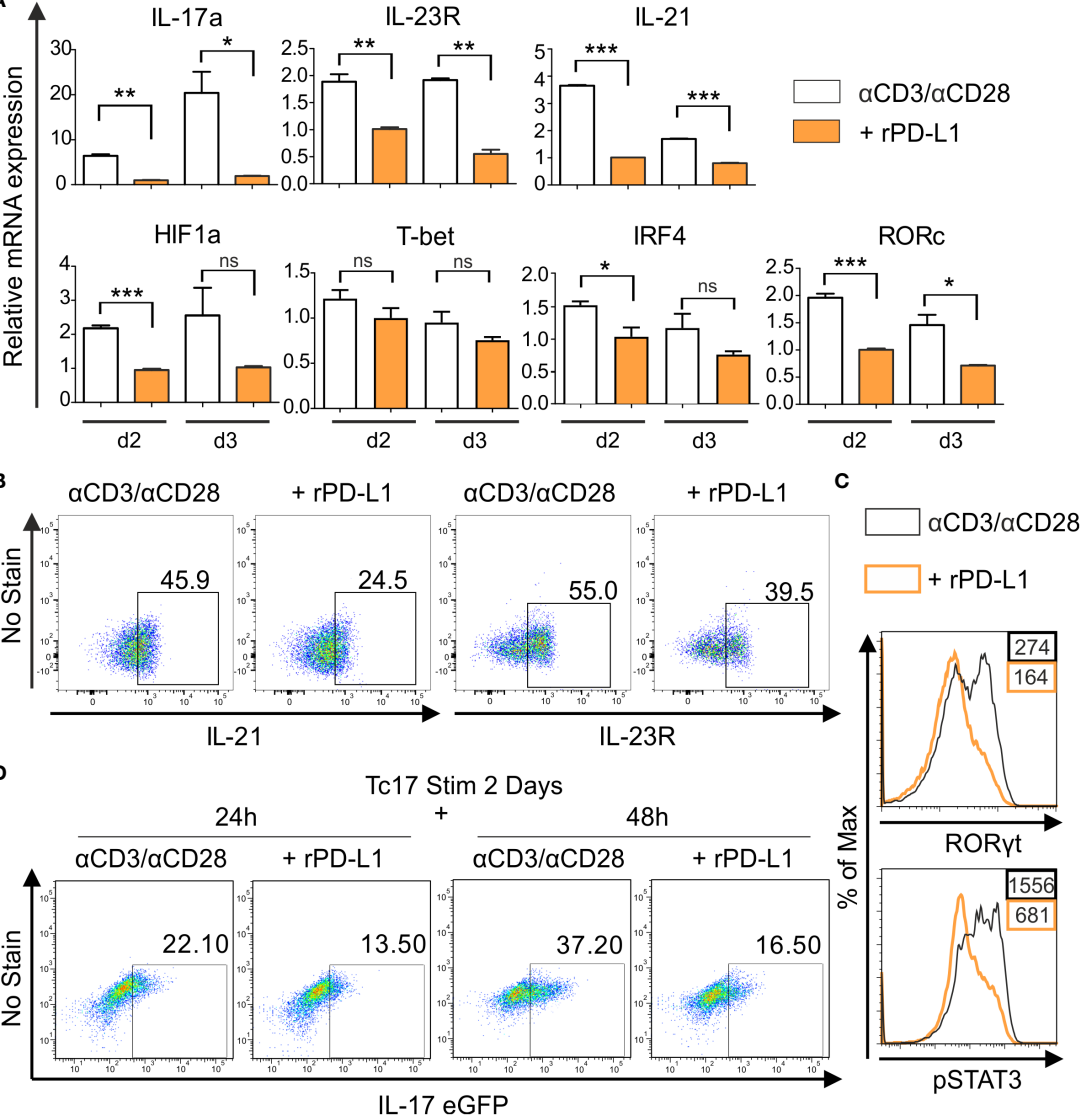
PD-1 suppresses Tc17 hallmarks. **(A)** CD8^+^ T-cells from C57BL/6 mice were stimulated as in (2C) and harvested at indicated time points. The harvested cells were lysed, RNA was extracted, and RT was used to synthesize cDNA. The relative expression levels of the indicated genes analyzed using real-time PCR are shown as mean+SEM of replicates from an experiment. **(B, C)** Tc17 cells were stimulated as in **(A)** and expression of IL-21 and IL-23R **(B)**, RORγt and pSTAT3 **(C)** was measured by flow cytometry 3 days after activation. **(D)** CD8^+^ T-cells from IL-17A-eGFP reporter mice were stimulated with microspheres immobilized with anti-CD3, anti-CD28 for 2 days under Tc17 conditions. These cells were then engaged with microspheres immobilized with rPD-L1 or not, associated with anti-CD3 and anti-CD28 for indicated time points and thereafter IL-17 expression was measured by flow cytometry. The data is representative of two to three independent experiments. ***P<0.001, **P<0.01, *P<0.05; ns, not significant, calculated by unpaired *t-*test.

To further investigate the PD-1 regulated intrinsic mechanism underlying the distinct degrees of Tc17 programs, we performed a qPCR analysis to determine mRNA accumulation of Tc17 specific molecules. For this purpose, we used RNA extracted from Tc17 CD8^+^ T-cells that were stimulated with anti-CD3, anti-CD28 with IgG control or with rPD-L1 (**Figure 2C**). As CD8^+^ T-cells can cycle cytokine production on and off (49), all stimulated CD8^+^ T-cells with or without PD-1 signaling under Tc17 conditions were included in the analysis to determine the effect of PD-1 on mRNA expression. Intriguingly, the Tc17 cells stimulated in the absence of PD-1 signaling demonstrated higher levels of mRNA expression of Tc17-supporting factors RORc, IRF4, HIF-1a, IL-21 and IL-23R at day two after stimulation (**Figure 3A**) but not of T-bet (**Figure 3A**). To stress physiological relevance of Tc17-specific accumulation of mRNAs, we also detected increased IL-21, IL-23R and RORγt protein expression by flow cytometry in 3 day stimulated Tc17 cells in the absence of rPD-L1 crosslinking (**Figures 3B, C**). Next, phosphorylated STAT3, which is known to be a central transcription factor in Tc17 differentiation and is able to enhance IL-17 transcription on its own as well as other Tc17 supporting factors (19), was analyzed. Enhanced frequencies of pSTAT3 expressing Tc17 cells were detected in the absence of PD-1-engagement (**Figure 3C**). Together, Tc17 cells showed an upregulation of Tc17‐ program supporting molecules in the absence of PD-1 engagement, and this might represent a higher degree of Tc17 differentiation in these cells compared with Tc17 cells getting PD-1 engagement.

To determine if PD-1 transmits an inhibitory signal on Tc17 differentiation only during early activation or also have additional functions on established Tc17 cells, a model including pre-stimulated Tc17 cells generated from CD8^+^ T-cells of IL-17A-eGFP reporter mice was applied. In this model CD8^+^ T-cells from IL-17A-eGFP reporter mice were initially stimulated with anti-CD3, anti-CD28 antibodies immobilized on microspheres in a Tc17-like microenvironment (IL-23, IL-6, TGFβ, see *Materials and methods*). Two days later, pre stimulated CD8^+^ T-cells were triggered in a recall response with anti-CD3, anti-CD28 with or without rPD-L1 immobilized on microspheres. When analyzing IL-17A-eGFP expressing cells 48h after beginning of the recall response, Tc17 cells in the absence of PD-1 engagement displayed a further enhanced increase in the frequency of IL-17A-eGFP expressing cells compared to stimulations providing PD-1 engagement (**Figure 3D**). These results indicated that PD-1 signaling provides a strong inhibitory signal on Tc17 differentiation, not only during primary stimulation, but also on Tc17 cells that show an already running Tc17 program.

### PD-1 suppresses lineage plasticity and anti-tumor potential of Tc17 cells

**Figure 4:**
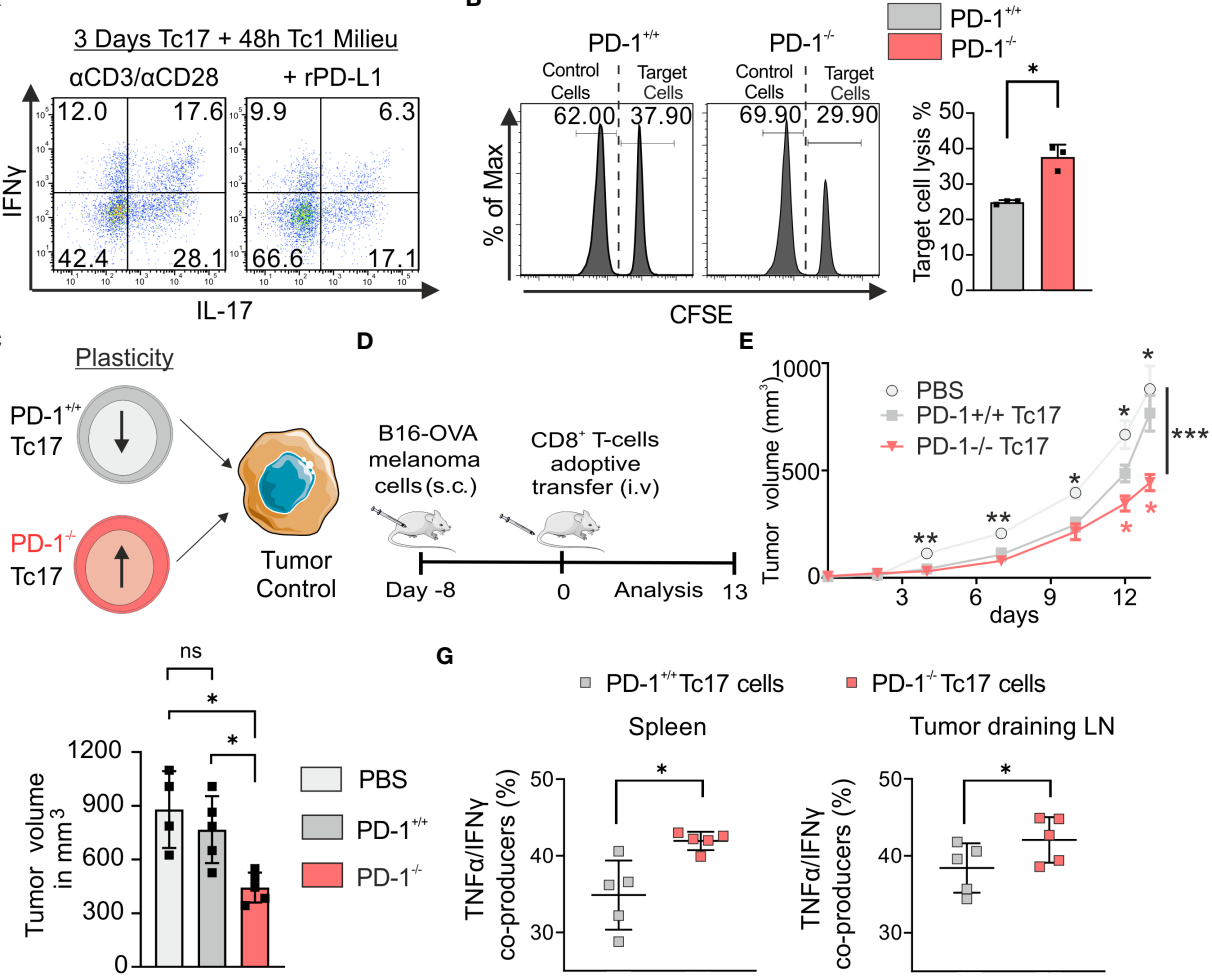
PD-1 limits plasticity and anti-tumor potential of Tc17 cells. **(A)** CD8^+^ T-cells from C57BL/6 mice were stimulated with microspheres immobilized with anti-CD3, anti-CD28 for 3 days under Tc17 conditions. These cells were then restimulated with fresh microspheres immobilized with anti-CD3, anti-CD28, and rPD-L1 or IgG under Tc1 conditions for 48h and thereafter IL-17 and IFN-γ expression was measured by flow cytometry. **(B)** Naïve PD-1^+/+^ and PD-1^-/-^ OT-1 CD8^+^ T cells were cultured under Tc17 conditions for 3 days as in **Figure 1**. T-cell depleted splenocytes were stained separately with different concentrations of CFSE (5 μM and 0.25 μM) and named accordingly as CFSE^high^ and CFSE^low^. Only the CFSE^high^ cells were loaded with OVA_257–264_ peptide and were considered as target cells. CFSE^low^ and CFSE^high^ cells (target cells) were pooled together at 1:1 ratio and a mixture of these cells were added to the pre-differentiated CD8^+^ T cells (effector cells). After 24 hours of restimulation, CFSE labelled cells were detected and quantified by flow cytometry and target cell lysis was calculated and presented in bar graph. **(C)** Determination of PD-1^+/+^ and PD-1^-/-^ Tc17 cells efficiency in tumor control **(D)** Schematic of the tumor experiment model. Recipient Ly5.1 mice were s.c. injected with B16-OVA melanoma cells. 8 d later, when a visible tumor was present, PBS or PD-1^+/+^ or PD-1^-/-^ OT-1 CD8^+^ T cells that had been stimulated under Tc17 conditions for 3 d were adoptively transferred into the tumor bearing mice through intravenous (i.v.) injection, and tumor growth was measured for the following days. The **(C, D)** were partly generated using Servier Medical Art, provided by Servier, licensed under a Creative Commons Attribution 3.0 unported license. **(E)** Curves showing mean tumor growth ± SEM with time in days for each treatment group (n=4-5). **(F)** Tumor volume in the recipient mice on day 13 after transfer of PBS or Tc17 cells. **(G)** Adoptively transferred CD45.2^+^ cells were surface stained ex vivo in the single cell suspensions from spleen and tumor draining lymph nodes of the tumor-bearing mice on day 13, and were analyzed for TNF-α, and IFN-γ production by flow cytometry. Data points represent individual mice with mean ± SD. ***P<0.001, **P<0.01, *P<0.05; ns, not significant, calculated by two-way RM ANOVA followed by Holm-Sidak’s multiple comparisons test for each individual time point [(E: black asterisks: PBS vs PD-1^-/-^ Tc17, red asterisks: PD-1^-/-^ Tc17 vs PD-1^+/+^ Tc17), F] or Welch’s *t*-test **(B, G)**.

Tc17 cells are known to be plastic in nature and have ability to convert to Tc1 like cells (18). To determine if PD-1 regulates plasticity of Tc17 cells, a model was applied, in which downstream targets of the Tc1 lineage (IFN‐γ) were analyzed in Tc17 cells re-stimulated in Tc1-type cytokine milieu, with or without rPD-L1 immobilized on microspheres. Intriguingly, re-stimulated Tc17 cells in the absence of rPD-L1 crosslinking displayed enhanced plasticity with increased IFN‐γ and IFN‐γ/IL-17 co-producers expression (**Figure 4A**). Considering the suppressive effect of rPD-L1 crosslinking on plasticity of re-stimulated Tc17 cells to Tc1 like cells, we next investigated the cytotoxic potential of PD-1^+/+^ and PD-1^−/−^ OT-1 Tc17 cells by performing an *in vitro* cytotoxicity assay using OVA_257–264_ labeled T-cell‐depleted splenocytes (target cells). In support of enhanced Tc17 plasticity in the absence of rPD-L1-crosslinking an increase in target cell‐specific lysis by PD-1^−/−^ Tc17 cells was observed in comparison to PD-1^+/+^ Tc17 cells (**Figure 4B**). Together these results indicated that PD-1 signaling suppresses Tc17 plasticity and ability to convert toward a Tc1‐like phenotype upon re-stimulation thereby reducing their cytotoxic potential.

To explore the functional mechanism of Tc17 cells with varying lineage plasticity with and without PD-1 signaling, we determined the ability of PD-1^+/+^ and PD-1^-/-^ type 17–skewed cells to promote antitumor immunity (**Figure 4C**) *in vivo*. For this we have adoptively transferred equal number of all cultured PD-1^+/+^ or PD-1^-/-^ OT-1 Tc17 cells into mice with pre-established melanoma (by B16 OVA-expressing melanoma cells) in a mouse model. Recipient tumor-bearing mice were used on a Ly5.1 background to differentiate adoptively transferred congenic OT-1 CD45.2 CD8^+^ T-cells. Progression of the established tumor was measured for up to 13 d following adoptive T-cell transfer (**Figure 4D**). In PBS-treated tumor-bearing mice, tumor outgrowth was progressing uncontrolled dramatically. In tumor-bearing mice adoptively transferred with Tc17 cells, tumor growth measurements clearly showed that despite enhanced IL-17 expression before adoptive transfer, PD-1^−/−^ Tc17 cells controlled tumor progression, in comparison to that of PD-1^+/+^ Tc17 cells (**Figures 4E, F**). To address PD-1 regulated plasticity *in vivo*, adoptively transferred Tc17 cells (CD45.2^+^), were identified in the single cell suspensions of spleen and tumor draining lymph nodes (dLN) from tumor bearing mice at the end of the experiment and analyzed *ex vivo*. Indeed, in contrast to primary stimulation of Tc17 cells before adoptive transfer, they had changed their cytokine profile: Both PD-1^+/+^ or PD-1^−/−^ Tc17 cells lost their IL‐17^+^ phenotype to a large extent and displayed enhanced expression of Tc1-like characteristics. Intriguingly, in comparison to PD-1^+/+^, PD-1^−/−^ Tc17 cells displayed a significant increase in polyfunctional IFN‐γ/TNF‐α co- producing cells *ex vivo* (**Figure 4G**). These kind of double producers are well known for anti-tumor immunity (50) and correlate well with reduced melanoma progression in mice adoptively transferred with PD-1^−/−^ Tc17 cells. Together, these results indicate that absence of PD-1 signals profoundly augments the antitumor activity of Tc17 cells.

### PD-1 limits cytotoxic potential of Tc17 cells by impinging stemness associated molecules

**Figure 5:**
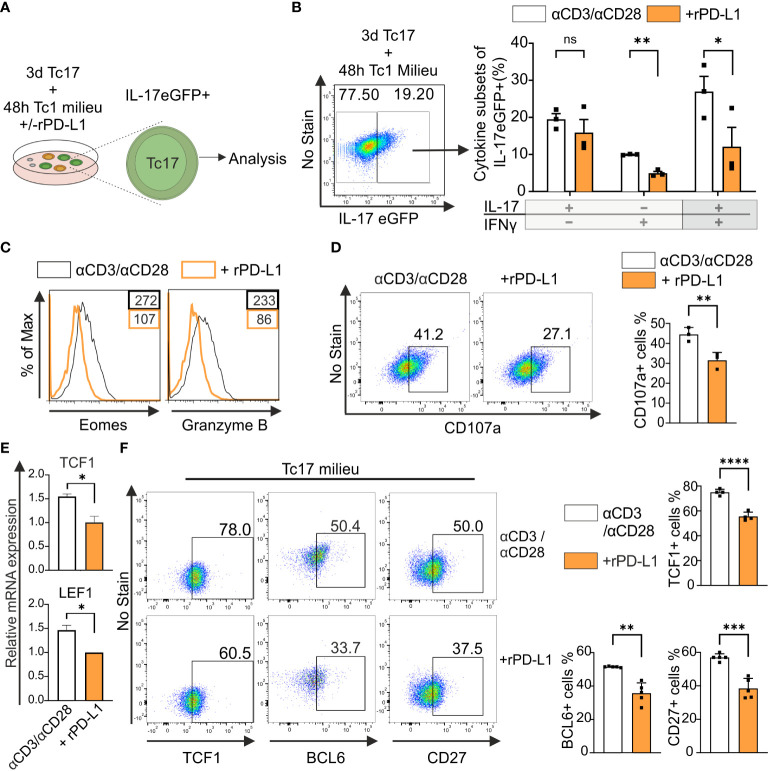
PD-1 suppresses cytotoxic potential and stemness of Tc17 cells. **(A, B)** CD8^+^ T-cells from IL-17A-eGFP reporter mice were stimulated with microspheres immobilized with anti-CD3, anti-CD28 for 3 days under Tc17 conditions. These cells were then restimulated with fresh microspheres immobilized with anti-CD3, anti-CD28, and rPD-L1 or IgG under Tc1 conditions for 48h and thereafter Tc17 cell plasticity **(A)** was determined by measuring IL-17 and IFN-γ expression **(B)** by flow cytometry within IL-17 eGFP expressing previous IL-17 producers. The **(A)** was partly generated using Servier Medical Art, provided by Servier, licensed under a Creative Commons Attribution 3.0 unported license. **(C)** CD8^+^ T-cells were stimulated as in **(A)** and, Eomes and granzyme B expression was measured by flow cytometry. **(D)** CD8^+^ T-cells from C57BL/6 mice were stimulated as in **(A)** and analyzed for the expression of degranulation-associated surface molecule CD107a. **(E)** CD8^+^ T-cells from C57BL/6 mice were stimulated with microspheres immobilized with anti-CD3, anti-CD28 and rPD-L1 (+rPD-L1) or IgG (αCD3/αCD28) antibodies under Tc17 condition and harvested 24h after activation. The harvested cells were lysed, RNA was extracted, and RT was used to synthesize cDNA. The relative expression levels of TCF1 and LEF1 genes analyzed using real-time PCR are shown as mean+SD of replicates from an experiment. **(F)** Tc17 cells were stimulated as in **(E)** and expression of TCF1, BCL6 and CD27 were measured by flow cytometry at day 3. The data is representative of two to five independent experiments. Data points represent individual experiments with mean+SD. ****P < 0.0001, ***P<0.001, **P<0.01, *P<0.05; ns, not significant, calculated by Welch’s *t-*test.

Considering the above reported strong anti-tumor immunity by PD-1^−/−^ Tc17 cells, we hypothesized – that the lack of PD-1 signaling mediates by default conversion of IL-17-producing cells to Tc1-like cells when stimulated in Tc1-type cytokine environment. IL-17A-eGFP expressing CD8^+^ T-cells were used to determine transcriptional plasticity in well-differentiated IL-17 producers (**Figure 5A**). Flow cytometric analysis showed that upon recall response in Tc1-type cytokine milieu, eGFP+ CD8^+^ T-cells displayed enhanced Tc1 lineage plasticity. However, in the absence of rPD-L1-crosslinking, a further increase in the frequency of IFN‐γ and IL-17/IFN‐γ‐coproducing cells was detected (**Figure 5B**). Additionally, Tc1 supporting transcription factor Eomes and cytotoxic molecule granzyme B expression were enhanced in the absence of rPD-L1-crosslinking (**Figure 5C**). In addition, the degranulation marker CD107a expression was also enhanced in the absence of rPD-L1-crosslinking (**Figure 5D**), further strengthening that PD-1 signaling suppresses Tc17 plasticity towards Tc1 like cells thereby reducing their cytotoxic potential.

Recent research identified TCF1^+^ tumor infiltrating lymphocytes with stem-cell like properties are associated with improved antitumor immunity and response to immunotherapy (51). IL-17-producing T-cells display stem-cell like properties including enhanced persistence, multipotency and self-renewal along with high expression of TCF1 (52). To determine the impact of PD-1 on TCF1 expression in Tc17 cells, we performed a qPCR analysis to determine mRNA accumulation. For this purpose, we used RNA extracted from the whole setting of either CD8^+^ T-cells that were stimulated with anti-CD3, anti-CD28 with rPD-L1 or counterparts stimulated with anti-CD3, anti-CD28 and IgG1 control in the presence of IL-6, IL-23 and TGFβ. (**Figure 5E**). Intriguingly, in line with enhanced Tc17 differentiation, the control Tc17 cells in the absence of PD-1 signaling demonstrated significantly higher levels of mRNA expression of TCF1 and downstream molecules - surrogate markers for stemness - LEF1 and BCL6 (**Figure 5E**, data not shown). In support of relevance of mRNA expression, we also detected increased frequencies of TCF1 and BCL6 protein expressing CD8^+^ T-cells stimulated under Tc17 condition in the absence of rPD-L1 crosslinking (**Figure 5F**). Using iMFI for analysis gave similar results (data not shown). CD27^+^ IL-17-producing T-cells have been shown to exhibit higher expression levels of TCF1, and indeed IL-17-producing T-cells encompassing CD27^+^TCF1^hi^ subset have been inferred with stemness-associated features (53). In line with enhanced expression of TCF1, CD27 expressing CD8^+^ T-cells stimulated under Tc17 condition were enhanced in the absence of PD-1 engagement (**Figure 5F**). Together these results further indicated that PD-1 signaling impinges on stemness associated features of Tc17 cells and therby reduce lineage plasticity and cytotoxic potential that supports antitumor immunity.

## Discussion

An underestimated aspect of the inhibitory receptor PD-1 is its high expression on IL-17-producing Tc17 cells that are only weakly cytotoxic, whereas most studies focused on its expression specifically on IFNγ-producing highly cytotoxic Tc1 cells. Here, our data reveal that PD-1 negatively regulates Tc17 differentiation and increases the resistance of Tc17 plasticity. In light of PD-1 blockade for tumor therapy, genetically inactivated PD-1^-/-^ OT-1 CD8^+^ T-cells showed an augmented capacity to enhance Tc17 differentiation *in vitro* in a T helper type 17 (Th17)-like cytokine environment and mount immunity to tumor tissue in an IFNγ dependent manner in tumor bearing mice. We further demonstrate that PD-1 limits stem cell like properties of Tc17 cells thereby suppressing their ability to convert to highly cytotoxic Tc1-like cells. Ultimately, this study provides new evidence that PD-1 acts as an inhibitory receptor, not only during the differentiation of IFNγ-producing Tc1 cells (37), but in particular during differentiation of IL-17-producing Tc17 cells as well. Important for tumor rejection, it goes far beyond being a simple inhibitor of Tc17 cells as it also limits plasticity of Tc17 cells.

The work presented here demonstrates that PD-1 inhibits IL-17 expression when CD8^+^ T-cells are primed in a Th17-like cytokine environment. Intriguingly, IL-17 expression was specifically inhibited by cell-intrinsic PD-1-signalling in CD8^+^ T-cells. Despite the fact that IFNγ is known to inhibit differentiation of IL-17-producing T-cells (39), a marginally enhanced IFN‐γ expression by genetic inactivation of PD-1 had no dampening effect on enhanced Tc17 differentiation by these cells (**Figure 1A**). The cell-cell interaction of CD8^+^ T-cells with APCs is superior of the impact of soluble factors and has been shown for other potent co-stimulatory molecules (54). In the same line, the suppressive effect of PD-1 on IL-17 expression was consistent in co-cultures of PD-1 expressing and PD-1 genetically inactivated Tc17 cells (**Figure 2B**). It has been reported that ligand for PD-1, PD-L1, is expressed not only on APCs but also on T-cells and restrains differentiation of IL-17-producing CD4^+^ T-cells (44). However, this suppression of CD8^+^ T-cells was not detectable as PD-1^-/-^ Tc17 cells displayed enhanced Tc17 differentiation irrespective of PD-L1 surface expression on T-cells. In addition, fraternal suppression is neglectable, as anti-CD3 and anti-CD28 engaged Tc17 cells show enhanced IL-17 production compared to anti-CD3, anti-CD28 and PD-1-engagment.

As our study shows that PD-1 is rapidly expressed upon activation and is able to inhibit low affinity antigen-triggered stimulation, it may well be able to restrain T-cells that would not normally be activated. As, tumor antigens might often rather be low affinity antigens due to negative selection of autoantigens, PD-1 will limit the antigen-specificities of the responding T-cell pool (55). Restraining reponses against low affinity antigens has been shown for CTLA-4 as well. As both checkpoint inhibitors are quite effective - albeit mechanistically in different ways (56), this may be the general mechanism to aim for in tumor therapy.

Previous reports point towards the idea that transcriptional profile of Tc1 and Tc17 cells oppose each other’s differentiation program (57, 58). It has been well recognized that PD-1 suppresses secretion of Tc1 cytokines (38). However, despite suppression of Tc1 transcriptional profile PD-1 signaling does not enhance differentiation of IL-17-producing CD8^+^ T-cells even when stimulated with IL-17 inducing cytokines. Moreover, PD-1 signaling reduced expression of Tc17 supporting molecules (**Figures 3A–C**) and even inhibits pre-established Tc17 cells. Therefore, PD-1-mediated signaling likely impinges at a common upstream molecule such as IRF4 (59). This also makes PD-1 to a central regulator in inhibiting differentiation of Tc17 cells along with Tc1 cells.

IL-17-producing T-cells are reported for their capacity to self-renew, persist in time and differentiate into IFN-*γ*-producing cells (60), making them interesting potential candidates for new T-cell-based therapies. In chronic infection models and tumor samples it has been demonstrated that, persistent progenitor exhausted TCF1^high^ CD8^+^ T-cells recall and give rise to cytotoxic short lived terminally exhausted TCF1^low^ cells required for effective immunity against infections and cancers (61). Accordingly, increased expression of TCF1 has been reported in Tc17 cells (52). Enhanced expression of TCF1 in Tc17 cells lacking PD-1 signaling (**Figure 5F**) indicates that strong PD-1 signaling in Tc17 cells may promote differentiation of more exhausted TCF1^low^ cells, while a weaker PD-1 signal during primary stimulation of Tc17 cells may promote differentiation of less exhausted TCF1^high^ cells that may recall better. In addition, costimulatory receptor CD27 expression was also enhanced in Tc17 cells lacking PD-1 signaling (**Figure 5F**), which further supports that these cells might have enhanced stemness potential to convert to Tc1 like cells, as T-cells encompassing CD27^+^TCF1^hi^ subset have been inferred with stemness associated features (53). Moreover, along with TCF1 an enhanced expression of BCL6 is also observed in Tc17 cells lacking PD-1 signaling, indicating this axis of TCF1-BCL6 may not only repress T-cell exhaustion and enhance stemness but also improves persistence of IL-17-producing CD8^+^ T-cells (62).

*In vivo*, PD-1^+/+^ Tc17 cells displayed an impaired potential in controlling tumor progression. Strikingly, PD-1^-/-^ Tc17 cells despite having an enhanced Tc17 transcriptionary mechanism restored the ability to control tumor progression (**Figure 4E**). Tc17 cells are known to be phenotypically unstable (18). Indeed, adoptively transferred Tc17 cells gave rise to an increased number of IFN-γ/TNF-α co-producers, which correlates well with tumor rejection (50). Intriguingly, PD-1^-/-^ Tc17 cells gave rise to increased number of IFN-γ/TNF-α co-producers in comparison to PD-1^+/+^ Tc17 cells (**Figure 4G**), further supporting TCF1 data that these cells have high stemness potential. IL-17A-eGFP expressing Tc17 cells upon re-stimulation under Tc1 cytokine milieu indicated that Tc1-like IFN-γ-producing cells are transitioned also from former IL-17-producing cells and this transition is enhanced in the absence of PD-1 signaling (**Figure 5A**). Along with enhanced IFN-γ production, absence of PD-1 signaling also displayed a significantly enhanced expression of otherwise repressed Eomes, granzyme B and CD107a expression, correlating well with enhanced cytotoxic activity and anti-tumor potential (2, 63). Enhanced Eomes expression in the absence of PD-1 signaling shows that IL-21 in Tc17 cells is not able to repress Eomes anymore (64). Furthermore, enhanced IL-23R was also not able to stabilize enhanced Tc17 differentiation in the absence of PD-1 signaling. However, a Tc17 program was still functional in the cells lacking PD-1 signaling as they continued to express IL-17 along with IFN-γ upon re-stimulation in a Tc1 cytokine milieu. As Tc1-like cells keep characteristics of Tc17 cells, such as longevity (19), a continued Tc17 program in Tc1-like cells may help in increasing their persistence and provide long-term protection against tumors. Therefore, in terms of tumor therapy, PD-1 blockade might be a double edged sword, on one hand enhancing Tc17 differentiation that might limit tumor rejection, on the other hand promoting switch of established Tc17 cells into Tc1 cells with longevity that drive tumor rejection. These results might explain tumor rejection versus hyperprogressive disease in some cancer patients treated with PD-1 inhibitor (65).

PD-1 blockade immunotherapy is now widely used in the treatment of several solid tumors. A majority of PD-1 blockade effects are studied on IFN-γ-producing Tc1 lymphocytes that with their high effector capacity are considered to be crucial to mediate rapid elimination of tumor cells. However, these cells are short living in comparison to Tc17 cells. In this instance, our finding that PD-1 blockade can also modulate differentiation and plasticity of long living IL-17-producing CD8^+^ T-cells provides central insights into long term effects of PD-1 blockade against tumors. Our data also suggest that PD-1-mediated cell intrinsic effects are distinct from that of CTLA-4, thus, blockade of both molecules might be of advantage to initate tumor rejection as well as longevity of the executing cells (56).

## Data availability statement

The original contributions presented in the study are included in the article/**Supplementary Material**. Further inquiries can be directed to the corresponding author.

## Ethics statement

The animal study was reviewed and approved by Landesverwaltungsamt Sachsen-Anhalt (No. 42502-2-1533 UniMD).

## Author contributions

MB-W and AA designed the study. AA performed the experiments, interpreted the findings and analyzed data. AA and MB-W wrote the manuscript. HL and MP have been involved in the study and critically read the manuscript. MB-W acquired funding and supervised the study. All authors contributed to the article and approved the submitted version.
