# Supplementary material for: PD-1 limits differentiation and plasticity of Tc17 cells
Source: Front Immunol. 2023 Apr 28;14:1104730. doi: 10.3389/fimmu.2023.1104730 (PMC10186197; doi:10.3389/fimmu.2023.1104730)
Supplement: Supplementary file 1 [file DataSheet_1.pdf]

Supplementary Information:

Table S1. List of antibodies used for FACS analysis

Antibody	Clone	Catalog number	Manufacturer
anti-CD8α	53–6.7	100706	Biolegend
anti-PD-1	29F.1A12	135210	Biolegend
anti-CD25	PC61	102020	Biolegend
anti-CD44	IM7	103040	Biolegend
anti-IL-6Rα	D7715A7	115812	Biolegend
anti-PD-L1	10F.9G2	124308	Biolegend
anti-IL-17	Tc11–18H10.1	506916	Biolegend
anti-IL-4	11B11	504104	Biolegend
anti-IL-10	JES5-16E3	505026	Biolegend
anti-IL-2	JES6-5H4	503808	Biolegend
anti-CD69	H1.2F3	104512	Biolegend
anti-CD27	LG.3A10	124226	Biolegend
anti-CD107a	1D4B	121625, 121606	Biolegend
anti-IL-23R	12B2B64	150903	Biolegend
anti-IFNγ	XMG1.2	25-7311-82	Thermo Fisher Scientific
anti-CD44	IM7	25-0441-81	Thermo Fisher Scientific
anti-CTLA-4	UC10-4B9	17-1522-82	Thermo Fisher Scientific
anti-IL-21	mhalx21	12-7213-82	Thermo Fisher Scientific
anti-Eomes	Dan11mag	12-4875-80	Thermo Fisher Scientific
anti-TNFα	MP6-XT22	48-7321-82	Thermo Fisher Scientific
anti-Granzyme B	GB12	MHGB04	Thermo Fisher Scientific
anti-RORγt	AFKJS-9	12-6988-82	Thermo Fisher Scientific
anti-ICOS	15F9	12-9940-81	ebioscience
anti-BCL6	K112-91	561525	BD Biosciences
anti-TCF1	S33-966	566692	BD Biosciences
anti-CD45.2	104	553772	BD Biosciences
anti-pSTAT3 (Rabbit mAb)	D3A7	9145	Cell signaling
donkey anti-rabbit IgG	Poly4064	406414	Biolegend

Table S2: Primer pairs used in quantitative real-time PCR

RORc	Forward: 5´-TGC AAG ACT CAT CGA CAA GG-3´<br>Reverse : 5´-AGG GGA TTC AAC ATC AGT GC-3´
BCL6	Forward: 5´- GGG ACA TCT TGA CGG ACG TT-3´<br>Reverse: 5´- TCA CGG GGA GGT TTA AGT GC-3´
GAPDH	Forward: 5´-GTC CCG TAG ACA AAA TGG TG-3´<br>Reverse: 5´-CAA GCT TCC CAT TCT CGG -3´
TCF7	Forward: 5´-GAC ATC AGC CAG AAG CAA G-3´<br>Reverse: 5´-GTG CTG TCT ATA TCC GCA G-3´
T-bet	Forward: 5´-TCA GGA CTA GGC GAA GGA GA-3´<br>Reverse : 5´-TAG TGG GCA CCT TCC AAT TC-3´
HIF1a	Forward: 5´-CGG CGA AGC AAA GAG TCT G-3´<br>Reverse : 5´-ATA ACT GAT GGT GAG CCT CAT AAC-3´
IRF4	Forward: 5´-TCT TCA AGG CTT GGG CAT TG-3´<br>Reverse : 5´-CAC ATC GTA ATC TTG TCT TCC AAG TAG-3´
IL-23R	Forward: 5´-TGA AAG AGA CCC TAC ATC CCT TGA-3´<br>Reverse : 5´-CAG AAA ATT GGA AGT TGG GAT ATG TT-3´
IL-21	Forward: 5´-CAT CAT TGA CCT CGT GGC CC-3´<br>Reverse : 5´-ATC GTA CTT CTC CAC TTG CAA TCC C-3´
IL-17a	Forward: 5´-CTC CAG AAG GCC CTC AGA CTA-3´<br>Reverse : 5´-AGC TTT CCC TCC GCA TTG ACA-3´

Figure S1

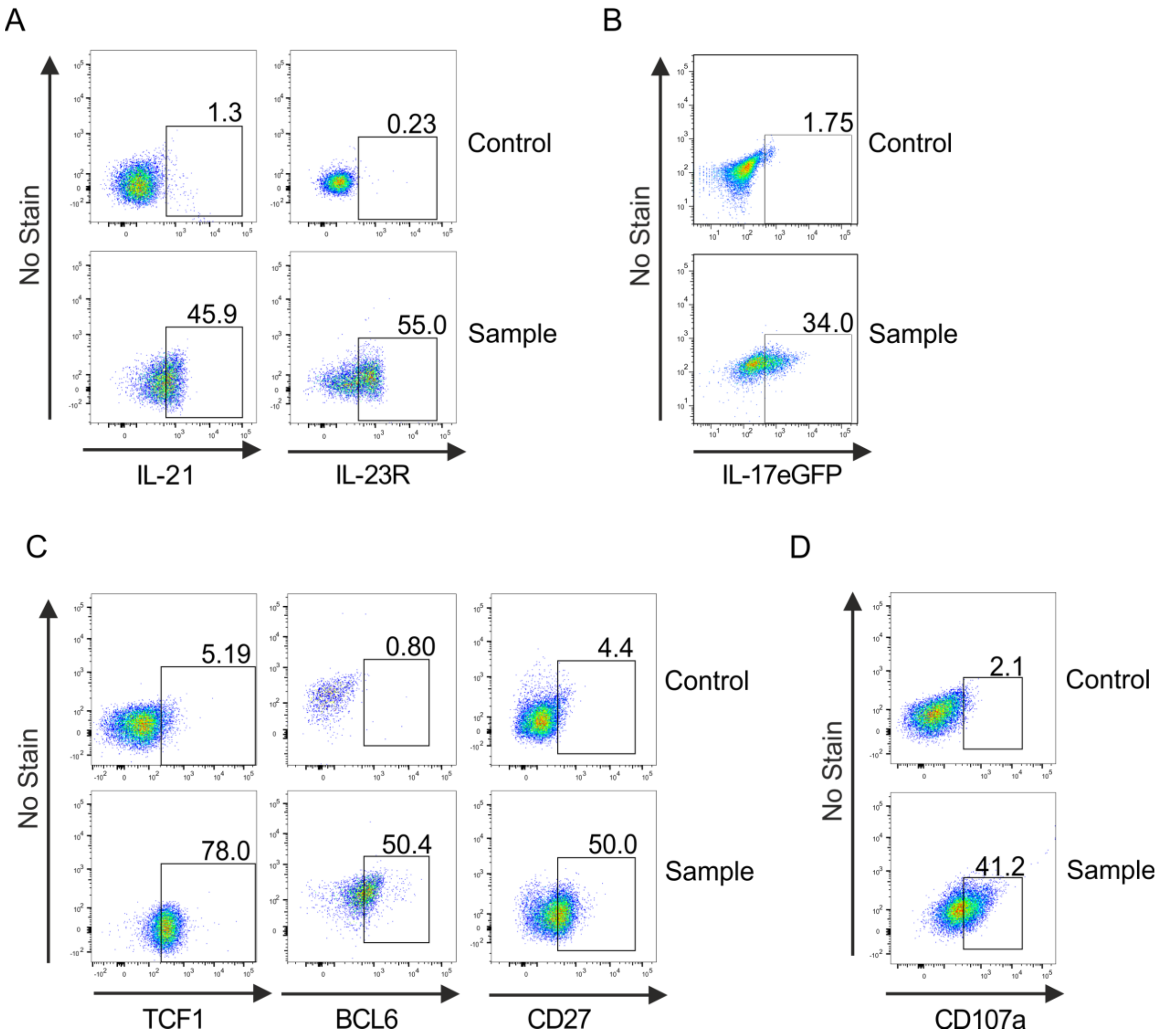

Figure S1. Gating strategy of flow cytometric dot plots.

(A,C,D) Unstimulated or unstained $CD8^+$ T-cells were used as controls to set the gate for IL-21, IL-23R, TCF1, BCL6, CD27 and CD107a staining in $CD8^+$ T-cells stimulated with microspheres immobilized with anti-CD3, anti-CD28 (Sample). (B) $CD8^+$ T-cells from C57BL/6 mice stimulated under Tc17 condition with anti-CD3, anti-CD28 were used as a control for IL-17eGFP gating in similarly stimulated $CD8^+$ T-cells (sample) from IL-17A-eGFP reporter mice.
